# Disseminated *Klebsiella pneumoniae* Infection Causing Multisystem Abscesses and Necrotising Fasciitis in an Immunocompetent Patient: A Case Report

**DOI:** 10.1155/crdi/5938248

**Published:** 2026-10-28

**Authors:** Qi Yi, Ying Huang, Huanxin Cao, Xingwen Zhang

**Affiliations:** ^1^ Department of Emergency Medicine Hunan Provincial People’s Hospital (The First Affiliated Hospital of Hunan Normal University) Changsha Hunan China hunnu.edu.cn

**Keywords:** *Klebsiella pneumoniae*, liver abscess, necrotising fasciitis, sepsis, source control

## Abstract

**Background:**

Disseminated *Klebsiella pneumoniae* infection that concurrently produces a liver abscess, bilateral pulmonary abscesses and necrotising fasciitis is uncommon in patients without overt immunocompromise. Definitive designation of a hypervirulent strain requires the string test and virulence‐gene testing, which are not always available at the point of care.

**Case Presentation:**

A previously healthy 48‐year‐old man, in whom a contaminated lower limb wound was the suspected portal of entry, subsequently developed a liver abscess, multiple bilateral pulmonary abscesses, necrotising soft‐tissue infection of the left lower limb and sepsis with circulatory instability. Culture of the liver‐abscess aspirate yielded a mucoid *K. pneumoniae* that was susceptible to carbapenems, ceftazidime/avibactam, amikacin and levofloxacin, among other agents. Because neither the string test nor virulence genes (e.g., *rmpA*, *iucA*) were examined, the isolate was described only as having a clinically hypervirulent phenotype based on mucoid colony morphology and the multisite invasive pattern. *Stenotrophomonas maltophilia* and multidrug‐resistant *Acinetobacter baumannii* were recovered sequentially from bronchoalveolar lavage fluid and sputum and were interpreted, in context, as airway colonisation or ventilator‐associated tracheobronchitis. After multisite percutaneous drainage, repeated debridement with vacuum sealing drainage, staged antimicrobial therapy and organ support, the patient recovered and was discharged; at the 3‐month follow‐up, the lesions had largely resolved without recurrence.

**Conclusion:**

This case suggests that multisite metastatic abscesses in an immunocompetent individual should raise suspicion for invasive *K. pneumoniae* infection. Early, adequate source control together with rational antimicrobial stewardship appears central to a favourable outcome. The conclusions are limited by the single‐case design and the absence of molecular confirmation of hypervirulence.

## 1. Introduction

*Klebsiella pneumoniae* is a common gram‐negative opportunistic pathogen. According to virulence characteristics, clinical isolates are broadly divided into classical *K. pneumoniae* (cKP) and hypervirulent *K. pneumoniae* (hvKP), but the two are not rigidly dichotomous categories; the convergence of virulence and resistance determinants is increasingly blurring this boundary [1–3]. In general, cKP infection occurs mainly in hospitalised patients with underlying conditions such as diabetes mellitus, alcohol misuse or immunosuppression and is predominantly healthcare‐associated. A hallmark of hvKP, by contrast, is its capacity to cause invasive infection in immunocompetent individuals, readily producing metastatic lesions at multiple sites—including liver abscess, pulmonary abscess, endophthalmitis and necrotising fasciitis—with strong disseminating capacity and rapid progression [1–4]. It should be noted that hvKP can likewise infect patients with diabetes or other comorbidities; infection of otherwise healthy people is an important feature rather than the sole defining criterion [3].

In recent years, reports of invasive *K. pneumoniae* infection have increased, and some cases progress rapidly to sepsis and multiple‐organ dysfunction, posing considerable diagnostic and therapeutic challenges [4, 5]. Here, we describe a previously healthy middle‐aged man in whom a contaminated lower limb wound was the suspected portal of entry and who subsequently developed a liver abscess, multiple bilateral pulmonary abscesses and necrotising soft‐tissue infection of the left lower limb; culture of the liver‐abscess aspirate yielded mucoid *K. pneumoniae*, and multidrug‐resistant nonfermenters were recovered from airway specimens during the course. We discuss source control, antimicrobial strategy and the interpretation of airway isolates against the complete clinical trajectory. The string test and molecular detection of the virulence genes *rmpA*, *iucA*, *iroB* and *magA* were not performed in this case; the presumptive diagnosis rests only on mucoid colony morphology and the pattern of multisite invasive infection, and this limitation is addressed in the Discussion section.

## 2. Case Presentation

### 2.1. Clinical History and Presentation

The patient was a 48‐year‐old man with no known chronic disease, history of infectious disease or use of immunosuppressive medication. Approximately 1 month before admission, he fell from a field ridge into a rice paddy, sustaining a contused laceration of the left lower limb with local swelling and pain; the wound was heavily soiled with mud and did not receive standardised debridement. Fever developed 18 days before admission, peaking at 39°C; empirical antimicrobial therapy at a local county hospital (specific regimen not documented) did not relieve the symptoms.

Ten days before admission (11 October 2024), he was transferred to Xiangxi Prefecture People’s Hospital, where incision and drainage of a left‐thigh abscess was performed, with 200–300 mL of yellowish‐brown pus drained daily. He received meropenem combined with linezolid (the outside‐hospital orders were not transferred with the record, so the exact doses are incompletely documented), and on 12 October 2024, he was endotracheally intubated and placed on invasive mechanical ventilation for respiratory failure. A blood culture obtained at the outside hospital grew *K. pneumoniae* (the original reports of abscess and sputum cultures were not transferred). His condition continued to deteriorate with worsening dyspnoea nine days before admission, and on 21 October 2024—defined as hospital Day 0—he was transferred to the emergency intensive care unit (ICU) of a provincial tertiary grade A hospital.

### 2.2. Admission Assessment

On physical examination on Day 0, the patient was sedated, intubated and mechanically ventilated (pressure support ventilation, FiO_2_ 50%, pressure support 12 cm H_2_O and positive end‐expiratory pressure 5 cm H_2_O). Temperature was 36.9°C, pulse 132 beats/min, respiratory rate 20 breaths/min and blood pressure 126/67 mmHg (maintained with norepinephrine 0.013 μg/kg/min). Mild bilateral scleral icterus was present; the pupils were equal and round at 3 mm with sluggish light reflexes. Breath sounds were coarse with scattered moist crackles; the heart rhythm was regular; the abdomen was distended with shifting dullness; approximately 6‐cm surgical incisions, packed with gauze and draining yellowish‐brown pus, were present over the lateral left thigh and lateral calf; and moderate pitting oedema involved both lower limbs. The Acute Physiology and Chronic Health Evaluation II (APACHE II) score was 16, with a predicted mortality of 25.6%, and the Sequential Organ Failure Assessment (SOFA) score was 3.

On Day 0, the white blood cell count was 12.50 × 10^9^/L, the neutrophil count 10.35 × 10^9^/L, haemoglobin 80 g/L and the platelet count 66 × 10^9^/L. Procalcitonin (PCT) was 0.28 ng/mL; C‐reactive protein (CRP) was 1.18 mg/L on Day 1 (22 October) and was below 0.5 mg/L on repeated subsequent testing, remaining within the normal range. Coagulation testing showed a D‐dimer of 11 040 ng/mL on Day 0, with a fibrinogen of 1.56 g/L on repeat testing on Day 1. Biochemistry showed albumin 26.82 g/L, sodium 150.6 mmol/L, myoglobin 470 ng/mL, triglycerides 4.41 mmol/L, high‐density‐lipoprotein cholesterol 0.34 mmol/L and glucose 7.08 mmol/L. Serial blood lactate was 2.1–2.8 mmol/L on Days 1–5, fell to 1.4 mmol/L on Day 7 and thereafter ranged from 1.6 to 2.5 mmol/L. The lowest blood pressure during admission was 94/46 mmHg (mean arterial pressure approximately 62 mmHg) on Day 4, and the low‐dose norepinephrine was gradually withdrawn on Days 9–10 after fluid resuscitation, plasma transfusion and source control. The admission glucose of 7.08 mmol/L was considered most consistent with stress hyperglycaemia; glucose ranged from 6.0 to 9.5 mmol/L during admission. There was no history of diabetes, and glycated haemoglobin was not measured. HIV serology and immunoglobulin concentrations were not routinely obtained, which is a limitation of classifying the patient as immunocompetent.

Head, chest and whole‐abdominal CT on Day 0 demonstrated inflammation of the bilateral maxillary, sphenoid and ethmoid sinuses; multiple bilateral pulmonary infectious lesions with multiple pulmonary abscesses; a subpleural calcified focus in the right lower lobe; an abscess in the right posterior hepatic lobe; abdominopelvic effusion; a nasojejunal tube in situ and a small amount of gas within the bladder. Echocardiography showed tachycardia with left‐ventricular systolic function within the normal range. Duplex ultrasound of the lower limb deep veins showed no thrombosis at admission; bedside ultrasonography demonstrated the liver abscess, pulmonary abscess and multiple left lower limb abscesses.

### 2.3. Microbiological Investigations

The principal microbiological results are summarised in Table 1. A blood culture collected on Day 1 (22 October) showed gram‐negative bacilli on direct smear. On the same day, ultrasound‐guided aspiration of the liver abscess yielded grey‐white pus, and culture of the aspirate grew *Klebsiella pneumoniae* subsp. *pneumoniae* (mucoid variant). The isolate was susceptible to piperacillin/tazobactam, ceftazidime, ceftazidime/avibactam, cefepime, cefoperazone/sulbactam, imipenem, meropenem, amikacin, tobramycin, ciprofloxacin, levofloxacin, doxycycline, minocycline, tigecycline, colistin and trimethoprim/sulfamethoxazole, and intermediate to ampicillin and cefazolin. Bronchoalveolar lavage fluid (BALF) collected on Day 3 (24 October) and reported on Day 10 (31 October) grew *Stenotrophomonas maltophilia* (50%) and *Acinetobacter baumannii* (50%) from the same specimen. *S. maltophilia* was resistant to ceftazidime but susceptible to ticarcillin/clavulanate, cefoperazone/sulbactam, levofloxacin, minocycline and trimethoprim/sulfamethoxazole. *A. baumannii* was resistant to ticarcillin/clavulanate, piperacillin/tazobactam, ceftazidime, cefepime, imipenem, meropenem and ciprofloxacin; susceptible to tobramycin, doxycycline, minocycline, tigecycline, colistin and trimethoprim/sulfamethoxazole and intermediate to levofloxacin and amikacin. Ascitic‐fluid and catheter‐tip cultures were negative. Sputum culture on Day 13 (3 November) again yielded *S. maltophilia* and *A. baumannii*. A blood culture from the outside hospital had previously grown *K. pneumoniae*, but its identification method and susceptibility profile were not available. Bacterial identification used an automated system with conventional biochemical reactions; susceptibility was determined by broth microdilution or disk diffusion and interpreted according to CLSI criteria.

**TABLE 1:** Principal microbiological investigations during hospitalisation.

Specimen	Collection date (hospital day)	Pathogen	Summary of susceptibility/result
Blood culture	22 Oct (Day 1)	Gram‐negative bacilli (direct smear)	Gram‐negative bacilli on direct smear; final identification and susceptibility were not archived in the clinical record
Liver‐abscess aspirate	22 Oct (Day 1)	*K. pneumoniae* subsp. *pneumoniae* (mucoid)	Susceptible to carbapenems, ceftazidime/avibactam, cefoperazone/sulbactam, amikacin, levofloxacin and tigecycline; intermediate to ampicillin and cefazolin
BALF	Collected 24 Oct (Day 3); reported 31 Oct (Day 10)	*S. maltophilia* (50%) + *A. baumannii* (50%)	*S. maltophilia*: ceftazidime‐resistant; susceptible to cefoperazone/sulbactam, levofloxacin, minocycline and TMP‐SMX. *A. baumannii*: carbapenem‐resistant; susceptible to doxycycline, minocycline, tigecycline, colistin and TMP‐SMX
Ascitic fluid	24 Oct (Day 3)	Negative	—
Catheter tip	30 Oct (Day 9)	Negative	—
Sputum	3 Nov (Day 13)	*S. maltophilia* + *A. baumannii*	Susceptibility profiles identical to the BALF isolates

### 2.4. Clinical Course and Management

Key clinical events are shown in Table 2, and a comprehensive day‐by‐day summary of the entire clinical course—including operative notes, microbiological findings, imaging modalities and results, antimicrobial regimens, ventilatory support parameters, serial PCT, CRP and lactate concentrations, and peak body temperature at each time point—is provided in Supporting Table 1.

**TABLE 2:** Timeline of key clinical events.

Hospital day	Date	Key events
Day 0	21 Oct	Transferred to the emergency ICU; intubated on mechanical ventilation; norepinephrine for circulatory support; ceftazidime/avibactam 2.5 g IV q8 h + daptomycin 0.5 g IV once daily; APACHE II score 16
Day 1	22 Oct	Daptomycin stopped and replaced with amikacin 0.4 g IV q12 h; gram‐negative bacilli on blood‐culture smear; ultrasound‐guided percutaneous catheter drainage of the liver abscess; fibreoptic bronchoscopy with BAL
Day 2	23 Oct	Bedside incision, debridement and drainage of left‐thigh and left‐calf abscesses; enteral nutrition started
Day 3	24 Oct	Incision and drainage of a left‐hand dorsum abscess; abdominal paracentesis with catheter (orange, clear ascites); bitten endotracheal tube replaced; BALF sent for culture
Day 4	25 Oct	Lowest blood pressure 94/46 mmHg; liver‐abscess aspirate culture reported mucoid *K. pneumoniae* (broadly susceptible)
Day 7	28 Oct	Operating‐room chronic‐ulcer repair/debridement of the left lower limb under intravenous anaesthesia; vancomycin 0.5 g IV q6 h added post‐operatively
Day 8	29 Oct	Right peroneal‐vein thrombosis confirmed on ultrasound (dalteparin 3750 IU had already been started on 25 Oct for the markedly elevated D‐dimer and was continued); VSD applied to the left lower limb; transfusions of leucoreduced red cells 1.5 U, cryoprecipitate 6 U and fresh frozen plasma
Day 9	30 Oct	Vancomycin discontinued in the early morning; dalteparin increased to 7500 IU; right internal‐jugular catheter removed and the tip sent for culture (subsequently negative)
Day 10	31 Oct	BALF culture reported *S. maltophilia* + *A. baumannii* from the same specimen; repeat CT showed shrinking abscesses; vasopressors discontinued
Day 11	1 Nov	Ceftazidime/avibactam and amikacin stopped and therapy de‐escalated to cefoperazone/sulbactam 3 g IV q8 h; successfully weaned and extubated, then high‐flow nasal cannula oxygen
Day 13	3 Nov	Sputum again yielded *S. maltophilia* + *A. baumannii*; interpreted as colonisation, with no change to the regimen
Day 14	4 Nov	Liver‐abscess drainage catheter removed; VSD removed; left lower limb debridement and wound suture
Day 19	9 Nov	Clinically stable and discharged; continue IV cefoperazone/sulbactam for 2 weeks at the local hospital, followed by 1 month of an oral antibiotic selected according to susceptibility (total course approximately 2 months); rivaroxaban 20 mg orally once daily

#### 2.4.1. Source Control

On Day 1, ultrasound‐guided percutaneous catheter drainage of the liver abscess was performed, yielding grey‐white pus. On Day 2, bedside incision and debridement of the left‐thigh and left‐calf abscesses drained approximately 200 and 150 mL of pus, respectively. On Day 3, a left‐hand dorsum abscess was drained and an abdominal catheter was placed; the ascitic fluid was orange and relatively clear, and its culture was negative, a picture attributed to hypoalbuminaemia and sepsis‐related capillary leakage. On Day 7, operating‐room ulcer repair and debridement of the left lower limb were undertaken; vacuum sealing drainage (VSD) was applied on Day 8 (29 October). On Day 14, the liver‐abscess drainage catheter was removed, the VSD was dismantled and the left lower limb wounds were debrided and sutured. Intraoperatively, necrotic subcutaneous and fascial tissue of the left lower limb were evident; however, tissue was not submitted for histopathology, and no deep‐tissue culture was obtained, which is a shortcoming in the evidence supporting the diagnosis of necrotising fasciitis.

#### 2.4.2. Antimicrobial Therapy

The patient had received meropenem combined with linezolid at the outside hospital but continued to deteriorate. After transfer (Day 0), following antimicrobial‐stewardship review, and given multisite abscess formation, severe infection with circulatory instability and the possibility of gram‐positive coinfection of the left lower limb soft tissues, the initial regimen was ceftazidime/avibactam 2.5 g IV q8 h combined with daptomycin 0.5 g IV once daily. On Day 1, because inflammatory markers remained elevated and in light of the soft‐tissue infection, daptomycin was stopped and replaced with amikacin 0.4 g IV q12 h to broaden gram‐negative coverage while limiting the potential risks of daptomycin in severe sepsis. After the Day‐7 lower limb debridement, a transient rise in the peripheral white blood cell count (18.09 × 10^9^/L) prompted a short course of vancomycin 0.5 g IV q6 h, which was discontinued in the early morning of Day 9. Importantly, the liver‐abscess isolate was susceptible to both meropenem and imipenem (MIC ≤ 0.25 μg/mL for each), with no evidence of carbapenem resistance or KPC production; clinical deterioration during meropenem therapy at the outside hospital was, therefore, more likely attributable to inadequate source control—multiple undrained abscesses and nondebrided necrotic tissue—than to microbiological resistance. The choice of ceftazidime/avibactam after transfer was based on disease severity, prior treatment failure and the stewardship team’s judgement rather than on any evidence of resistance.

On Day 11, with normal temperature, a fall in PCT to 0.09 ng/mL, continuing improvement in inflammatory markers and radiological shrinkage of the abscesses, ceftazidime/avibactam and amikacin were stopped and therapy was deescalated, according to susceptibility, to cefoperazone/sulbactam 3 g IV q8 h. Renal function was monitored throughout, with no overt amikacin‐related nephrotoxicity. After discharge, IV cefoperazone/sulbactam was continued for 2 weeks at the local hospital, followed by 1 month of an oral susceptible agent; the overall antimicrobial course was approximately 2 months.

#### 2.4.3. Organ Support and Comprehensive Care

For respiratory support, the patient remained intubated on mechanical ventilation and underwent repeated fibreoptic bronchoscopy with BAL (approximately eight times in total); ventilator settings were gradually reduced, and he was successfully extubated on Day 11 and transitioned to heated humidified high‐flow nasal cannula oxygen. For circulatory support, low‐dose norepinephrine was required at admission, with a nadir blood pressure of 94/46 mmHg on Day 4; vasopressors were gradually withdrawn on Days 9–10 after fluid resuscitation, plasma transfusion and source control. Nutritional support combined short‐peptide enteral feeding by nasogastric/jejunal tube with parenteral nutrition and whey‐protein supplementation, with gradual transition to whole‐protein feeding. In total, approximately 3000 mL of fresh frozen plasma, 1.5 U of leucoreduced red blood cells and 10 U of cryoprecipitate were transfused to correct hypoalbuminaemia, anaemia and coagulopathy. In addition, dalteparin was started on 25 October (Day 4) at 3750 IU/day subcutaneously because of a markedly elevated D‐dimer; after a right peroneal‐vein thrombosis was confirmed on Day 8, anticoagulation was continued and increased to 7500 IU/day on Day 9, being used until 3 November; rivaroxaban 20 mg orally once daily was substituted at discharge.

### 2.5. Outcome and Follow‐Up

The peak temperature during admission was 38.7°C (Day 4, 25 October); after Day 7, the temperature was mostly normal, with only occasional low‐grade fever (peak ≤ 37.8°C). PCT declined progressively from 0.28 to 0.09 ng/mL (Day 11), blood lactate reached the normal range on Day 7, and ventilator support was gradually reduced. Repeat chest and abdominal CT showed progressive resolution of the pulmonary and hepatic abscesses. On Day 19, the patient was clinically stable with normal vital signs, no fever and satisfactory wound healing, and he was discharged.

As specified in the discharge plan, the left lower limb sutures were removed intermittently at the local hospital at approximately 2 weeks after surgery (around 18 November 2024). At the 3‐month follow‐up, repeat chest and abdominal CT showed that the abscesses had largely resolved, with no radiological evidence of liver cirrhosis or pulmonary fibrosis; the left lower limb wounds were well healed, without erythema or discharge, and there was no recurrence of infection. Because follow‐up was limited to 3 months, longer term complications continue to be observed.

## 3. Discussion

### 3.1. Recognition of Invasive *Klebsiella* Syndrome and the Limitation of Presuming Hypervirulence

This patient’s illness began with a local infection after a contaminated lower limb wound and was followed by liver abscess, multiple bilateral pulmonary abscesses and abscesses of the limbs, consistent with the clinical picture of invasive *Klebsiella* syndrome [4, 6, 7]. The liver‐abscess aspirate yielded mucoid *K. pneumoniae*, and the occurrence of metastatic suppurative lesions at multiple sites in an immunocompetent individual is suggestive of a hypervirulent phenotype. Nevertheless, a mucoid colony morphology is not equivalent to the hypermucoviscous phenotype (which requires a positive string test), and the hypermucoviscous phenotype is not itself equivalent to hypervirulence; a pattern of multisite invasive infection may suggest hvKP but cannot reliably distinguish hvKP from cKP or from hybrid strains [1–3, 8]. Because the string test and the virulence genes *rmpA/rmpA2*, *iucA*, *iroB* and *peg-344* were not examined, definitive evidence for hvKP is lacking; throughout this report, we, therefore, use the formulation “*K. pneumoniae* with a clinically hypervirulent phenotype” rather than labelling the isolate as hvKP. Regarding the portal of entry, although a temporal association exists between the contaminated lower limb wound and the subsequent hepatic and pulmonary abscesses, the direction of spread cannot be established: Haematogenous dissemination after traumatic inoculation is one possibility, but haematogenous spread from a primary liver abscess to soft tissue is likewise a classic pattern of invasive *Klebsiella* syndrome [4, 6]. We, therefore, describe the lower limb wound as the suspected portal and explicitly acknowledge the alternative.

### 3.2. The Role of Source Control in Severe Disseminated Infection

In sepsis, antimicrobial therapy and programmatic source control are equally important, and delayed source control is associated with increased mortality [9]. In this patient, a systematic source‐control programme was implemented after transfer: percutaneous catheter drainage of the liver abscess, incision and drainage of limb abscesses, drainage of ascitic fluid and debridement of necrotic tissue combined with VSD. For a liver abscess larger than 3 cm in diameter, ultrasound‐ or CT‐guided percutaneous catheter drainage is the standard treatment [10, 11]; for necrotising soft‐tissue infection, WSES guidelines stress avoiding delay to the first operation, undertaking thorough debridement once the diagnosis is established and frequently requiring repeat debridement [9]. Two lower limb debridements, on Days 7 and 14, illustrate the necessity of repeated source control. Of note, the diagnosis of necrotising fasciitis in this case rested principally on the fascial necrosis seen intraoperatively and on the clinical picture; histopathology and deep‐tissue culture were not performed, leaving the diagnostic evidence incomplete, and this limitation should be borne in mind when interpreting the case.

### 3.3. Antimicrobial Strategy and Stewardship

Empirical therapy for severe infection should be started promptly after microbiological specimens are obtained and should cover the most likely pathogens and resistance‐risk factors; as culture and susceptibility become available, timely deescalation reduces unnecessary broad‐spectrum exposure [9, 12, 13]. An important lesson here is that deterioration during meropenem therapy at the outside hospital does not equate to microbiological failure. The liver‐abscess isolate was highly susceptible to meropenem (MIC ≤ 0.25 μg/mL), and progression was more plausibly attributable to multiple undrained abscesses and nondebrided necrotic tissue [9]. Ceftazidime/avibactam after transfer was chosen for disease severity, prior failure and stewardship judgement rather than because of resistance; the addition of daptomycin (later replaced by amikacin) reflected concern over mixed soft‐tissue infection. On Day 11, once the pathogen was defined and inflammatory markers were improving, timely deescalation to cefoperazone/sulbactam was undertaken, with renal monitoring during amikacin exposure, consistent with stewardship principles [12–14]. With respect to duration, invasive *K. pneumoniae* liver‐abscess syndrome generally requires four to 6 weeks or more of treatment, continued until the cavity has largely or completely resolved [11]. The approximately 2‐month course in this patient, with multisite abscesses and necrotising soft‐tissue infection, was commensurate with disease severity.

### 3.4. Interpretation of Airway Isolates and Inflammatory Markers

*S. maltophilia* and multidrug‐resistant *A. baumannii* were recovered sequentially from BALF (collected on Day 3 and reported on Day 10) and from sputum (Day 13). Both organisms appeared after more than 10 days of invasive mechanical ventilation and broad‐spectrum antibiotic exposure; over the same period, the systemic inflammatory markers declined steadily and the pulmonary lesions continued to resolve on imaging. No additional antimicrobial changes were made for these organisms, and the clinical condition continued to improve after bronchoscopic lavage and enhanced airway drainage. Taken together, the isolates are more consistent with airway colonisation or ventilator‐associated tracheobronchitis (VAT) than with a primary pathogen responsible for the sepsis [15, 16]. This judgement is retrospective and cannot be regarded as established: the BALF culture reported no quantitative or semiquantitative colony count, and the record does not document purulent secretions or a new pulmonary infiltrate, which are elements required for a diagnosis of VAP/VAT. Persistent recovery of the two organisms on later sputum culture also counsels caution about any claim that bacteria were “cleared by lavage”; a single negative culture would not suffice to demonstrate eradication. In addition, PCT (0.09–0.28 ng/mL) and CRP (1.18 mg/L on Day 1 and below 0.5 mg/L thereafter) remained low throughout the admission, seemingly disproportionate to the extensive abscess formation and to a severe clinical picture requiring vasopressors. Plausible explanations include prolonged broad‐spectrum therapy before admission, limited release of inflammatory cytokines into the circulation from fibrous‐walled foci and the timing of sampling. This illustrates that, in patients already receiving antimicrobials, normal or only mildly elevated inflammatory markers do not exclude severe invasive infection.

## 4. Conclusions

We describe a 48‐year‐old immunocompetent man in whom a contaminated lower limb wound was the suspected portal of entry and who subsequently developed liver abscess, multiple bilateral pulmonary abscesses and left lower limb necrotising soft‐tissue infection; culture of the liver‐abscess aspirate yielded a carbapenem‐susceptible mucoid *K. pneumoniae*, whose hypervirulent phenotype could only be presumed because virulence‐gene testing was not performed. After systematic source control (repeated abscess drainage and debridement of necrotic fascia with VSD), staged antimicrobial therapy and organ support, the infection was controlled and the patient was discharged, with near‐complete resolution of lesions and no recurrence at 3 months. This clinical course suggests that multisite metastatic abscesses should prompt suspicion of invasive *K. pneumoniae* even when inflammatory markers are low; that apparent antimicrobial failure should first prompt assessment of the adequacy of source control rather than being attributed simply to resistance; and that drug‐resistant nonfermenters isolated from the airway of a ventilated patient should be interpreted by integrating systemic inflammatory trends with serial imaging to distinguish colonisation from infection. As a single‐centre single‐case report lacking molecular confirmation of hvKP and histopathological evidence of necrotising fasciitis, generalisability of these conclusions is limited.

## Author Contributions

Qi Yi and Ying Huang conceived and designed the case report; Qi Yi collected and verified the clinical data and drafted the manuscript; Huanxin Cao and Xingwen Zhang contributed to data collection and patient management; Ying Huang critically reviewed and revised the manuscript and provided supervision.

## Funding

This work was supported by the Medical and Health Industry Joint Fund of the Hunan Provincial Natural Science Foundation (First Batch, 2026) (grant number 2026JJ81730), for the project entitled “Mechanism of S‐nitrosylation modification of Rac1 protein regulating neutrophil activity in acute pancreatitis‐associated lung injury” (2026–2028).

## Disclosure

All authors have read and approved the final manuscript.

## Consent

Written informed consent was obtained from the patient for publication of this case report and any accompanying clinical information.

## Conflicts of Interest

The authors declare no conflicts of interest.

## Supporting Information

Additional supporting information can be found online in the Supporting Information section.

## Supporting information


**Supporting Information 1** Supporting Table 1 provides a detailed chronological summary of the clinical course from 30 days before admission through discharge and the 3‐month follow‐up. For each time point, the table records clinical events and surgical procedures, operative notes, microbiological findings, imaging modality and results, the antimicrobial regimen, ventilatory support parameters, serial procalcitonin, C‐reactive protein and lactate concentrations, and peak body temperature; representative CT images are embedded within the table.

**Supporting Information 2** CARE.

## Data Availability

All data supporting the findings of this case report are included within the article and the accompanying supporting information.
